# Identification and Expression Analysis of the *CsMYB* Gene Family in Root Knot Nematode-Resistant and Susceptible Cucumbers

**DOI:** 10.3389/fgene.2020.550677

**Published:** 2020-12-03

**Authors:** Chunyan Cheng, Qingrong Li, Xing Wang, Ying Li, Chuntao Qian, Ji Li, Qunfeng Lou, Molly Jahn, Jinfeng Chen

**Affiliations:** ^1^College of Horticulture, Nanjing Agricultural University, Nanjing, China; ^2^Hebei University of Engineering, Handan, China; ^3^Nanjing Vegetable Science Research Institute, Nanjing, China; ^4^Jahn Research Group, Department of Agronomy, University of Wisconsin–Madison, Madison, WI, United States

**Keywords:** cucumber, resistant introgression line, MYB, root-knot nematode, resistant mechanism

## Abstract

MYB (myeloblastosis) transcription factors (TFs) play important roles in controlling various physiological processes in plants, such as responses to biotic and abiotic stress, metabolism, and defense. A previous study identified a gene, *Csa6G410090*, encoding a plant lipid transfer protein (LTP), as a possible regulator in cucumber (*Cucumis sativus* L.) of the resistance response to root-knot nematode (RKN) [*Meloidogyne incognita* Kofoid and White (Chitwood)]. Myb-type DNA-binding TFs were presumed to regulate downstream genes expression, including LTPs, however, the regulation mechanism remained unclear. To elucidate whether and which MYB TFs may be involved in regulation of the resistance response, this study identified 112 genes as candidate members of the *CsMYB* gene family by combining CDD and SMART databases, using the Hidden Markov Model (HMM) and manual calibration. Within this group, ten phylogenetic subgroups were resolved according to sequence-based classification, consistent with results from comprehensive investigation of gene structure, conserved motifs, chromosome locations, and *cis*-element analysis. Distribution and collinearity analysis indicated that amplification of the *CsMYB* gene family in cucumber has occurred mainly through tandem repeat events. Spatial gene expression analysis showed that 8 *CsMYB* genes were highly expressed at differing levels in ten different tissues or organs. The roots of RKN-resistant and susceptible cucumbers were inoculated with *M. incognita*, finding that *CsMYB* (*Csa6G538700*, *Csa1G021940*, and *Csa5G641610*) genes showed up-regulation coincident with upregulation of the “hub” gene *LTP* (*Csa6G410090*) previously implicated as a major gene in the resistance response to RKN in cucumber. Results of this study suggest hypotheses regarding the elements and regulation of the resistant response as well as possible RKN resistance-enhancing strategies in cucumber and perhaps more broadly in plants.

## Introduction

Myeloblastosis proteins, named for their discovery as transcription regulators in myeloblastosis, function in plants as widely distributed and functionally diverse plant genetic transcription factors (TFs). In plants, MYB TFs play crucial roles in regulating plant physiological and biochemical processes (e.g., Daniel et al., 1999; [Bibr B35][Bibr B62]; Sara et al., 2018). The MYB domain, consisting of a region of about 52 amino acids, bind DNA in a sequence-specific manner (Jin and Martin, 1999). The MYB gene family can be divided into four classes according to the number and type of MYB domains known as 1RMYB (or MYB-related), 2RMYB, 3RMYB, and 4RMYB, respectively. The 2RMYB class is specific to plants and the largest (Dubos et al., 2010), having been annotated genome-wide in many plants. Previous studies showed that there are 190 members of the 2RMYB family in *Arabidopsis* (Riechmann et al., 2000), 102 members in *Oryza sativa* (Chen et al., 2006), 117 members in *Vitis vinifera* (Matus et al., 2008), 192 members in *Populus trichocarpa* (Wilkins et al., 2009), 244 members in *Glycine max* (Du et al., 2012), and more than 200 members in *Zea mays* (Dias et al., 2003). Only 55 *2RMYB* genes, however, have been identified in the most recent version of cucumber genome sequence (*Cucumis sativus* L.), the smallest number identified in any plant species to date, owing to the absence of recent gene duplication events (Li et al., 2012).

Functions of these MYB TFs have been explored in many studies on plant defense and response to various biotic and abiotic stresses (Raffaele et al., 2008; Cominelli and Tonelli, 2009; Zhang et al., 2019; Wang H. H. et al., 2020). For example, *BOS1* in *Arabidopsis* encodes a *2RMYB* TF that regulates anther development and also responses to biotic stresses, such as biotrophic pathogens ([Bibr B38][Bibr B35]). *PbrMYB5* plays an active role in enhancing tolerance to chilling stresses in tobacco (*Nicotiana tabacum*) (Xing et al., 2019). *AtMYB60* and *AtMYB96* in *Arabidopsis* are required for ABA signaling, stomatal movement, drought stress and disease resistance (Cominelli et al., 2005; Seo et al., 2009, 2011; Seo and Park, 2010). In tomato (*Solanum lycopersicum* L.), R3-MYB inhibits anthocyanin production (Colanero et al., 2018). In kiwifruit (*Actinidia chinensis*), *MYB7* regulates transcriptional activation of metabolic pathway genes to modulate carotenoid and chlorophyll pigment accumulation (Ampomah-Dwamena et al., 2019). *McMYB10* is involved in anthocyanin biosynthesis in crabapple during continuous light treatments (Li et al., 2018). In cotton, *GhMYB7*, *GhMYB9*, and *GhMYB109* were involved in cotton fiber initial cells, as well as elongating fibers (Suo et al., 2003).

Although new insights regarding the mechanisms by which MYB proteins control various plant processes and several core genes have been identified, the TFs in question are usually encoded by very large multigene families confounding detailed mechanistic studies, consistent with the likelihood that many of the complex regulatory roles played by *MYB* genes-encoding-proteins remain unknown (Riechmann et al., 2000). Our previous comparative transcriptomic study showed that MYB TFs may participate in regulating the hub gene *LTP* (lipid transfer protein) of cucumber in resistance to root-knot nematode (RKN) [*Meloidogyne incognita* Kofoid and White (Chitwood)] (Wang et al., 2018). LTP, a member of plant pathogenesis-related protein (PRs), is involved in lipid transport in giant cells (GCs) membranes and receives signals stimulated by exposure to nematodes (Wang X. et al., 2020). These results from cucumber are consistent with results from *Arabidopsis*, where *LTP3* is positively regulated by the TF *MYB96* to mediate freezing and drought stress (Guo et al., 2013). However, the mechanism by which MYB TF(s) regulates LTP in cucumber is still unknown. In the present study, we carried out a systematic analysis of *CsMYB* genes in cucumber, showing that this family members are highly variable and apparently play a positive role in responding to *M. incognita* in cucumber. Our results provide more evidence regarding whether and how *CsMYB* genes regulate *LTP*, thereby potentially affecting resistance to RKN in cucumber.

## Materials and Methods

### Identification of Cucumber MYB Gene Family

The cucumber whole genome sequence was downloaded from the cucumber genome database^1^. The MYB binding domains (PF00249) in the sequence were identified and confirmed using the Hidden Markov Model (HMM) profile from PFam database^2^ applied as a query to previously annotated cucumber MYB-containing sequences, further identified by HMMSEARCH and manual screened using default parameter (*E* ≤ 1.2 × 10^–8^). HMMRESEARCH was applied to establish the cucumber MYB (*CsMYB*) gene family-specific model (Lozano et al., 2015), which was visualized online by SKylign^3^. Then all predicted cucumber MYB proteins with an E-value below 0.001 were selected using the cucumber MYB-specific HMM. The candidate genes for the *CsMYB* family were identified according to cucumber-specific MYB HMM.

All the candidate *CsMYB* genes initially obtained above were uploaded to BlastP by CDD (Conserved Domain Database) (Marchler-Bauer et al., 2017) and SMART (Simple Modular Architecture Research Tool) (Marchler-Bauer et al., 2015; Letunic and Bork, 2018) using the default parameter *E* ≤ 0.01. Four classes (1RMYB, 2RMYB, 3RMYB, and 4RMYB) of *CsMYB* genes were separated according to characteristic conserved domains within the MYB sequence.

All the predicted protein sequences of the candidate *CsMYB* family members were submitted to ExPASy^4^ to calculate the number of amino acids (aa), molecular weight (Mw) and isoelectric point (pI). The candidate *CsMYB* genes were renamed according to the repeat number of conserved domains and the physical position on the chromosome. The subcellular localization analysis of the candidate *CsMYB* family protein sequences were carried out by CELLO v.2.5 programme (subCELlular LOcalization predictor) at Molecular Bioinformatics Center^5^ (Yu et al., 2004, 2006).

### Gene Structure and Analysis of Conserved Motifs Among Cucumber MYB Gene Family Members

The exon and intron structures of the candidate *CsMYB* genes were obtained by comparison with the corresponding full-length genome sequences. Conserved motifs in the gene family were determined using the following parameters: arbitrary number of repetitions, up to 10; length, 6–200 amino acid residues via MEME software and predicted protein sequence (Bailey et al., 2009; Letunic et al., 2015). The gene structures and conserved motifs of *CsMYB* genes were displayed by TBTools (Chen et al., 2020).

### Sequence Alignment and Phylogenetic Analysis of *CsMYB*

The sequences of candidate *CsMYB* proteins obtained were used to create Clustal V multiple sequence alignment using MEGA7 with *AtMYBs* in *Arabidopsis thaliana*. MEGA7 was used to analyze the phylogeny and evolution of all the predicted protein sequences by using the adjacency method (neighbor-joining, NJ) criterion. According to the results of the alignment, all the nodes were analyzed by bootstrap under the parameters, No. of Differences, Uniform rates, Complete deletion, and then repeateds 1000 times. The maximum likelihood (maximum likelihood, ML) method was used to verify the phylogenetic tree. Besides, phylogenetic tree between cucumber and other species was constructed based on MYB gene sequences from Gcorn database^6^.

### Orthologous Analysis and Chromosome Localization

The candidate *CsMYB* genes were located on cucumber chromosomes by Mapchart (Voorrips, 2002). The duplication events within the gene family were analyzed by Multiple Collinearity Scan toolkit (MCScanX) (Wang et al., 2012) and Circos (Krzywinski et al., 2009). In order to reveal the homology of MYB gene between cucumber and *Arabidopsis thaliana*, the homology analysis of MYB gene between *Arabidopsis thaliana* and cucumber was constructed by using the Dual Synteny Plotter software.

### *Cis*-Elements Analysis and Prediction of CsMYB

The upstream sequence (1.5 kb) of *CsMYB* coding sequences were extracted from the cucumber genome sequence and submitted to PlantCARE^7^ (Magali et al., 2002) for identification of *cis*-elements. The Gene Structure Display Server (GSDS)^8^ (Li et al., 2012; Hu et al., 2014) is used to visualize all the regulatory elements involved in defense and stress response or defense factors, and to further analyze the regulatory elements related to resistance defense.

### Response to *Meloidogyne incognita*

In order to study changes in gene expression correlated with expression of resistance to *Meloidogyne incognita* in cucumber, gene expression in a RKN-resistant line, IL10-1, and RKN-susceptible line, “Beijingjietou” (CC3), that display marked differences in against *M*. *incognita* (Ye et al., 2011), were obtained directly from NCBI by SRP125669 (Wang et al., 2018) at 0, 1, 2 and 3 days after inoculation (dpi) with approximately 50 fresh and motile second-stage juveniles (J2) *M. incognita*. The readings per kilobyte million enzyme (RPKM) for each MYB gene were downloaded and calculated from NCBI^9^. The RPKM values were converted through log2 transformation value (Li et al., 2011), and the cucumber genome database HEATMAP was used to draw the heatmap online.

### RNA Isolation and RT-qPCR Analyses

Seeds of IL10-1 and CC3, provided by the State Key Lab of Cucurbit Genetics and Germplasm Enhancement of Nanjing Agricultural College, were surface sterilized (70% ethanol for 15 s and 1% sodium hypochlorite solution for 10 min, then rinsing with distilled water three times). Sterilized seeds were germinated on wet filter paper in a growth chamber at 28°C. After 3–4 days, seedlings were transplanted onto pluronic F-127 (Sigma-Aldrich, United States) gel to assess RKN infectivity (Wang et al., 2009), under controlled condition (28°C/16 h light, 24°C/8 h dark).

Inoculated root samples were harvested at 0, 1, 2, and 3 dpi as follows. Root tips from five different seedlings were mixed into a single biological sample, then 3 replicates were made from each sample. Total RNA from cucumber roots was extracted using the TRIzol reagent (Invitrogen, Carlsbad, CA, United States). Extracted RNA was quantified by Thermo NanoDrop 2000 (Thermo Fisher Scientific, MA, United States). cDNA was synthesized from the total RNA used for RNA-seq using the PrimeScript RT reagent Kit (Takara, Japan), and then qPCR, repeated three times, was performed on Bio-Rad CFX96 thermocycler using the 2 × SYBR green PCR master mix (Applied Biosystems). Seven DEGs were selected to verify the accuracy of the transcriptome results by qPCR. A housekeeping gene *Csa6M484600* encoding ACTIN was used as the internal control (Wilson et al., 2015). The primers for qPCR listed in Supplementary Table S3 were designed using Primer 6 software. The relative expression of each gene was calculated using the 2^–ΔΔ*Ct*^ method (Livak and Schmittgen, 2001). Each expression profile was independently verified in three replicated experiments performed under identical conditions. Primer sequences used are listed in Supplementary Table S3.

### Tissue-Specific Expression

In order to study the gene-specific expression of *CsMYB* genes in different tissues of cucumber, the serial number PRJNA80169 was used from NCBI to obtain cucumber RNA samples from different tissues and organs (leaves, stems, female flowers, male flowers, enlarged ovary (unfertilized), dilated ovary (fertilized), ovary, root, RNA-Seq data for tendrils and the base of tendrils (Harris et al., 2008).

## Results

### Identification of the Cucumber *CsMYB* Gene Family

A cucumber-specific model (Supplementary Figure S1) was built for typical MYB or MYB-like domains using the HMM profile (PF00249) of the MYB-conserved domain. A total of 197 *CsMYB* genes were selected from the cucumber genome database^10^. Combining the CDD and SMART databases using manual calibration, 215 genes were identified for the *CsMYB* gene family, further divided into the four subclasses according to the number of repeats of the conserved protein domain. Predicted CsMYB proteins (listed in Table 1) showed considerable redundancy, e.g., 33, 67, and 3 redundant proteins in the group MYB-related, R2R3-MYB, and R1R2R3-MYB, respectively. The number of unique proteins is 112, corresponding to the actual number of 112 genes.

**TABLE 1:** The characteristics of *CsMYB* -encoded proteins in cucumber.

**Rename**	**gene ID**	**Chr**	**length**	**MV (Da)**	**pI**	**Predicted location**	**Classification**
*Cs1RMYB-1*	Csa1G004120	1	89	10203.8	9.08	Extracellular	MYB_related
*Cs1RMYB-2*	Csa1G024160	1	248	27448.6	4.89	Nuclear	MYB_related
*Cs1RMYB-3*	Csa1G033200	1	280	31195.2	5.57	Nuclear	MYB_related
*Cs1RMYB-4*	Csa1G042350	1	252	27974.9	5.29	Nuclear	MYB_related
*Cs1RMYB-5*	Csa1G071840	1	313	34927.7	5.17	Nuclear	MYB_related
*Cs1RMYB-6*	Csa1G420880	1	299	33745.2	5.5	Nuclear	MYB_related
*Cs1RMYB-7*	Csa1G488750	1	103	11966.7	10.01	Chloroplast	MYB_related
*Cs1RMYB-8*	Csa2G007440	2	164	18840.4	4.34	Nuclear	MYB_related
*Cs1RMYB-9*	Csa2G035350	2	305	34400.3	7.44	Nuclear	MYB_related
*Cs1RMYB-10*	Csa2G049890	2	117	13463.8	9.42	Plasma Membrane	MYB_related
*Cs1RMYB-11*	Csa2G169770	2	160	18460.9	6.6	Cytoplasmic	MYB_related
*Cs1RMYB-12*	Csa2G229940	2	240	28644.2	10.61	Nuclear	MYB_related
*Cs1RMYB-13*	Csa2G229950	2	62	6881.8	8.55	Nuclear	MYB_related
*Cs1RMYB-14*	Csa2G352940	2	90	10047.6	9.89	Nuclear	MYB_related
*Cs1RMYB-15*	Csa2G370470	2	660	70029.6	8.11	Nuclear	MYB_related
*Cs1RMYB-16*	Csa3G045200	3	59	6782.7	9.69	Nuclear	MYB_related
*Cs1RMYB-17*	Csa3G122510	3	95	10392.6	5.2	Nuclear	MYB_related
*Cs1RMYB-18*	Csa3G134010	3	109	12886.9	10.84	Mitochondrial	MYB_related
*Cs1RMYB-19*	Csa3G135050	3	100	11579.5	10.09	Nuclear	MYB_related
*Cs1RMYB-20*	Csa3G165690	3	93	10737.2	9.34	Nuclear	MYB_related
*Cs1RMYB-21*	Csa3G740800	3	116	13176.1	9.07	Nuclear	MYB_related
*Cs1RMYB-22*	Csa3G742860	3	205	24306.1	6.94	Nuclear	MYB_related
*Cs1RMYB-23*	Csa3G828990	3	109	12185.8	9.32	Nuclear	MYB_related
*Cs1RMYB-24*	Csa3G848130	3	280	30712.4	9.85	Nuclear	MYB_related
*Cs1RMYB-25*	Csa3G891670	3	386	42250.2	8.67	Nuclear	MYB_related
*Cs1RMYB-26*	Csa4G000700	4	329	36786.4	8.62	Cytoplasmic	MYB_related
*Cs1RMYB-27*	Csa4G290190	4	674	76072.4	10.06	Nuclear	MYB_related
*Cs1RMYB-28*	Csa4G645320	4	51	5651.51	8.54	Nuclear	MYB_related
*Cs1RMYB-29*	Csa5G171690	5	109	12436.6	9.67	Mitochondrial	MYB_related
*Cs1RMYB-30*	Csa5G524690	5	158	17462.2	4.87	Nuclear	MYB_related
*Cs1RMYB-31*	Csa5G524720	5	83	9198.4	5.54	Plasma Membrane	MYB_related
*Cs1RMYB-32*	Csa6G105150	6	68	8080.3	11.56	Nuclear	MYB_related
*Cs1RMYB-33*	Csa6G136580	6	275	31613.5	4.44	Nuclear	MYB_related
*Cs1RMYB-34*	Csa6G303240	6	191	22007.6	4.85	Nuclear	MYB_related
*Cs1RMYB-35*	Csa6G311520	6	107	12124.7	9.75	Nuclear	MYB_related
*Cs1RMYB-36*	Csa6G495710	6	307	33757.5	10.25	Cytoplasmic	MYB_related
*Cs1RMYB-37*	Csa6G496960	6	56	6084	8.75	Cytoplasmic	MYB_related
*Cs1RMYB-38*	Csa6G499890	6	315	35014.2	6.03	Nuclear	MYB_related
*Cs1RMYB-39*	Csa7G179630	7	559	63088.6	6.91	Nuclear	MYB_related
*Cs2RMYB-1*	Csa1G004900	1	472	54883.7	6.3	Nuclear	R2R3-MYB
*Cs2RMYB-2*	Csa1G008430	1	368	41942.7	6.19	Nuclear	R2R3-MYB
*Cs2RMYB-3*	Csa1G009700	1	332	37603.3	9.14	Nuclear	R2R3-MYB
*Cs2RMYB-4*	Csa1G021940	1	308	33313	7.41	Nuclear	R2R3-MYB
*Cs2RMYB-5*	Csa1G029570	1	233	26589.2	8.84	Nuclear	R2R3-MYB
*Cs2RMYB-6*	Csa1G046820	1	378	42093.5	6.92	Nuclear	R2R3-MYB
*Cs2RMYB-7*	Csa1G109320	1	319	36229	8.2	Nuclear	R2R3-MYB
*Cs2RMYB-8*	Csa1G529120	1	432	48469.4	5.67	Nuclear	R2R3-MYB
*Cs2RMYB-9*	Csa1G561370	1	342	37757.4	5.94	Nuclear	R2R3-MYB
*Cs2RMYB-10*	Csa1G575180	1	249	27776.7	6.73	Nuclear	R2R3-MYB
*Cs2RMYB-11*	Csa1G682630	1	517	59414.2	6.97	Nuclear	R2R3-MYB
*Cs2RMYB-12*	Csa2G100550	2	265	29982.6	8.85	Nuclear	R2R3-MYB
*Cs2RMYB-13*	Csa2G174670	2	108	12650.5	10.2	Nuclear	R2R3-MYB
*Cs2RMYB-14*	Csa2G270220	2	232	26160.6	7.61	Nuclear	R2R3-MYB
*Cs2RMYB-15*	Csa2G302300	2	211	23544.3	10.95	Nuclear	R2R3-MYB
*Cs2RMYB-16*	Csa2G352410	2	205	23037	8.29	Nuclear	R2R3-MYB
*Cs2RMYB-17*	Csa2G355030	2	291	31804.4	9.57	Nuclear	R2R3-MYB
*Cs2RMYB-18*	Csa2G360620	2	243	27199.1	9.27	Nuclear	R2R3-MYB
*Cs2RMYB-19*	Csa2G427310	2	302	32296.8	8.13	Nuclear	R2R3-MYB
*Cs2RMYB-20*	Csa2G439230	2	300	33891.8	6.37	Nuclear	R2R3-MYB
*Cs2RMYB-21*	Csa3G076520	3	520	57033.1	7.78	Nuclear	R2R3-MYB
*Cs2RMYB-22*	Csa3G168940	3	306	33693.1	5.69	Nuclear	R2R3-MYB
*Cs2RMYB-23*	Csa3G182040	3	300	34029.5	10.12	Nuclear	R2R3-MYB
*Cs2RMYB-24*	Csa3G199590	3	220	25251.4	6.78	Nuclear	R2R3-MYB
*Cs2RMYB-25*	Csa3G264750	3	355	40501.9	9.6	Nuclear	R2R3-MYB
*Cs2RMYB-26*	Csa3G303630	3	268	30096.4	5.81	Nuclear	R2R3-MYB
*Cs2RMYB-27*	Csa3G386830	3	170	19348.4	4.48	Nuclear	R2R3-MYB
*Cs2RMYB-28*	Csa3G535090	3	225	25603.6	7.86	Nuclear	R2R3-MYB
*Cs2RMYB-29*	Csa3G592130	3	220	24887.1	6.61	Nuclear	R2R3-MYB
*Cs2RMYB-30*	Csa3G732480	3	269	30010.8	9.76	Extracellular	R2R3-MYB
*Cs2RMYB-31*	Csa3G812750	3	349	39131.2	9.07	Nuclear	R2R3-MYB
*Cs2RMYB-32*	Csa3G816030	3	295	32662.3	6.53	Nuclear	R2R3-MYB
*Cs2RMYB-33*	Csa3G824850	3	234	26130.2	9.77	Nuclear	R2R3-MYB
*Cs2RMYB-34*	Csa3G826690	3	314	35125.9	6.35	Nuclear	R2R3-MYB
*Cs2RMYB-35*	Csa3G901090	3	105	12050.7	8.76	Nuclear	R2R3-MYB
*Cs2RMYB-36*	Csa3G914600	3	254	28517.6	9.38	Nuclear	R2R3-MYB
*Cs2RMYB-37*	Csa4G022940	4	315	36057.3	6.51	Nuclear	R2R3-MYB
*Cs2RMYB-38*	Csa4G305350	4	378	40500	5.31	Nuclear	R2R3-MYB
*Cs2RMYB-39*	Csa4G308500	4	300	34358.2	6.81	Nuclear	R2R3-MYB
*Cs2RMYB-40*	Csa4G638510	4	230	25192.6	9.56	Nuclear	R2R3-MYB
*Cs2RMYB-41*	Csa4G641690	4	264	29140.4	8.35	Nuclear	R2R3-MYB
*Cs2RMYB-42*	Csa5G148680	5	261	28837.7	4.74	Nuclear	R2R3-MYB
*Cs2RMYB-43*	Csa5G152790	5	324	36414.6	6.44	Nuclear	R2R3-MYB
*Cs2RMYB-44*	Csa5G160120	5	259	29457.7	6.17	Nuclear	R2R3-MYB
*Cs2RMYB-45*	Csa5G198240	5	296	33799.6	8.84	Mitochondrial	R2R3-MYB
*Cs2RMYB-46*	Csa5G209490	5	285	31234.3	6.88	Nuclear	R2R3-MYB
*Cs2RMYB-47*	Csa5G353650	5	296	31215.5	6.92	Nuclear	R2R3-MYB
*Cs2RMYB-48*	Csa5G425400	5	301	33770.6	5.11	Nuclear	R2R3-MYB
*Cs2RMYB-49*	Csa5G579040	5	287	30876.7	6.17	Nuclear	R2R3-MYB
*Cs2RMYB-50*	Csa5G604410	5	465	52285.8	7.27	Nuclear	R2R3-MYB
*Cs2RMYB-51*	Csa5G605730	5	370	41397.2	7.08	Nuclear	R2R3-MYB
*Cs2RMYB-52*	Csa5G637660	5	399	44517.7	6.99	Nuclear	R2R3-MYB
*Cs2RMYB-53*	Csa5G641610	5	231	27272.2	9.77	Nuclear	R2R3-MYB
*Cs2RMYB-54*	Csa5G651640	5	295	33129	8.51	Nuclear	R2R3-MYB
*Cs2RMYB-55*	Csa6G040640	6	287	32771	6.87	Nuclear	R2R3-MYB
*Cs2RMYB-56*	Csa6G046240	6	234	26439.4	8.87	Nuclear	R2R3-MYB
*Cs2RMYB-57*	Csa6G046460	6	149	16821.1	7.68	Cytoplasmic	R2R3-MYB
*Cs2RMYB-58*	Csa6G121970	6	270	31333.9	6.92	Nuclear	R2R3-MYB
*Cs2RMYB-59*	Csa6G187960	6	316	35118.2	9.91	Nuclear	R2R3-MYB
*Cs2RMYB-60*	Csa6G344210	6	310	35826.2	9.56	Nuclear	R2R3-MYB
*Cs2RMYB-61*	Csa6G491690	6	302	32449.4	9.71	Nuclear	R2R3-MYB
*Cs2RMYB-62*	Csa6G495660	6	240	26841.1	10.28	Nuclear	R2R3-MYB
*Cs2RMYB-63*	Csa6G538700	6	290	33192	6.95	Nuclear	R2R3-MYB
*Cs2RMYB-64*	Csa7G043580	7	501	54622.8	6.31	Nuclear	R2R3-MYB
*Cs2RMYB-65*	Csa7G045590	7	255	29446.4	5.21	Nuclear	R2R3-MYB
*Cs2RMYB-66*	Csa7G046120	7	338	37544.7	6.39	Nuclear	R2R3-MYB
*Cs2RMYB-67*	Csa7G375780	7	255	29393.9	4.78	Nuclear	R2R3-MYB
*Cs2RMYB-68*	Csa7G413890	7	277	31884.7	4.97	Nuclear	R2R3-MYB
*Cs2RMYB-69*	Csa7G431360	7	1503	169045	4.87	Nuclear	R2R3-MYB
*Cs3RMYB-1*	Csa2G375240	2	1000	111544	5.54	Nuclear	R1R2R3-MYB
*Cs3RMYB-2*	Csa3G734230	3	551	61163.7	8.53	Nuclear	R1R2R3-MYB
*Cs3RMYB-3*	Csa3G889150	3	961	105806	5.3	Nuclear	R1R2R3-MYB
*Cs4RMYB*	Csa3G113280	3	1005	113684	7.06	Nuclear	4RMYB

Predicted physicochemical properties of the *CsMYB* gene family proteins, including molecular weight (M_W_), isoelectric point (pI) and length (aa), were analyzed through ExPASy online. *CsMYB* genes encoded proteins ranging from 56 to 1503 aa in length, pI values ranging from 4.34 to 11.56 and molecular weights from 6084 to 169044.6 Da. Protein subcellular location was predicted through CELLO program (v.2.5). It was found that the vast majority genes (89.72%) encoded proteins that were predicted to be intranuclear, while others were predicted to be located in mitochondria, chloroplasts, cytoplasm, and the plasma membrane (Table 1). Members of the *CsMYB* gene family were then renamed according to their classification (subclasses 1–4) and their chromosome location (*Cs1RMYB-1* to *Cs1RMYB-39*, *Cs2RMYB-1* to *Cs2RMYB-69*, *Cs3RMYB-1* to *Cs3RMYB-3*, and *Cs4RMYB*).

### Evolutionary Relationships, Gene Structure and Conserved Motif Analyses of the *CsMYB* Gene Family in Cucumber

**FIGURE 1:**
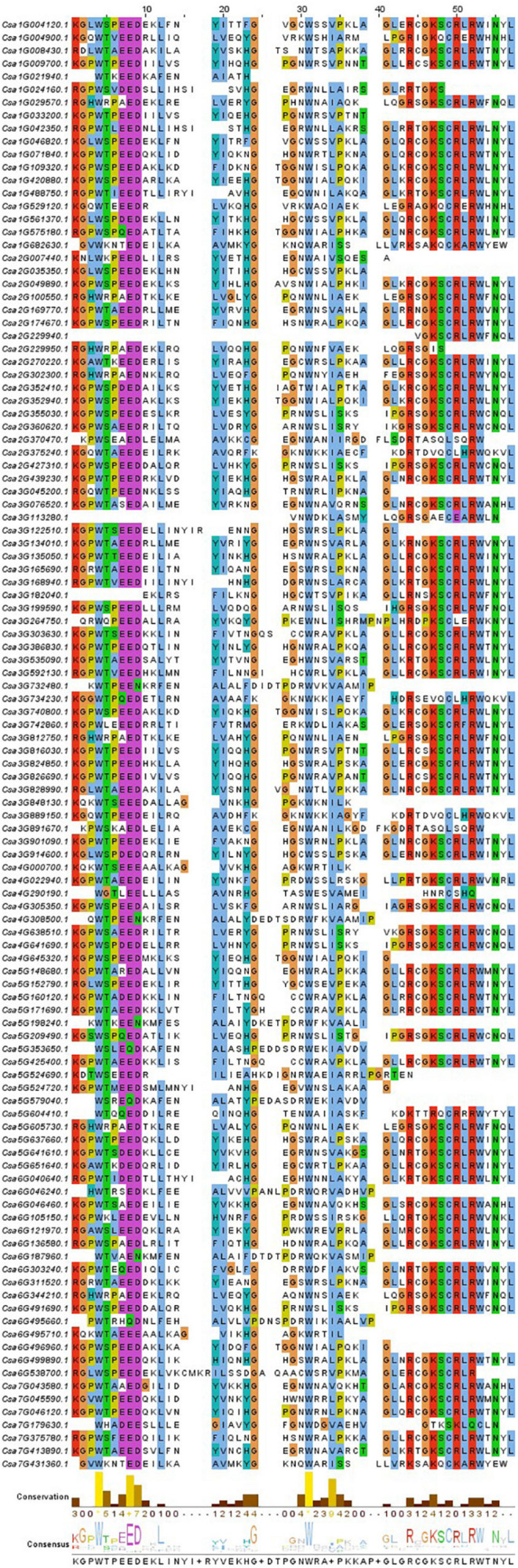
Alignment, degree of conservation and consensus of the conserved domain of *CsMYB* genes. The sequence logos of the cucumber special model was built based on full-length alignments of the MYB-conserved domain. The overall height of each stack indicates the conservation of the sequence at that position, whereas the height of letters within each stack represents the relative frequency of the corresponding amino acid.

**FIGURE 2:**
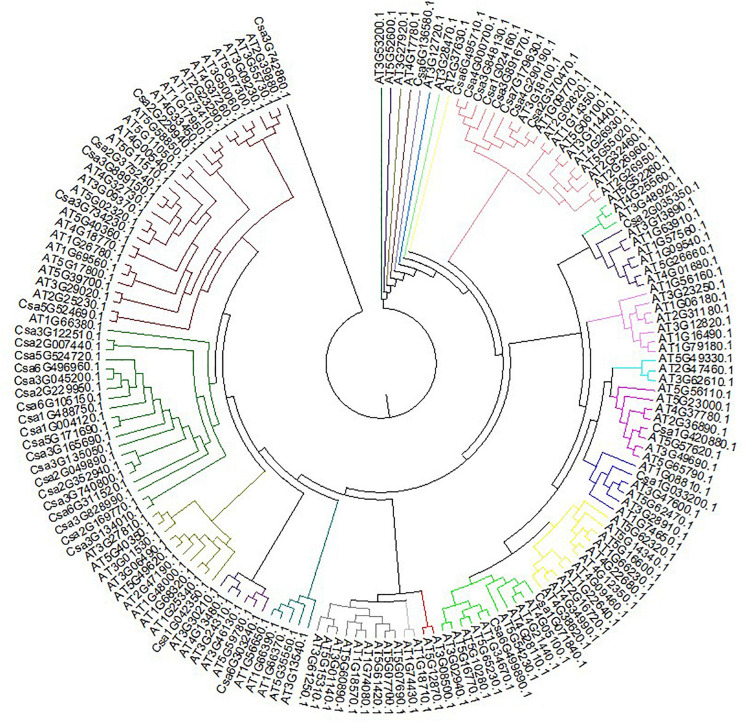
Phylogenetic relationships between *CsMYB* genes in cucumber and *AtMYB* genes in *Arabidopsis.* CsMYB proteins in were divided into ten groups based on sequence similarity grouped by color.

**FIGURE 3:**
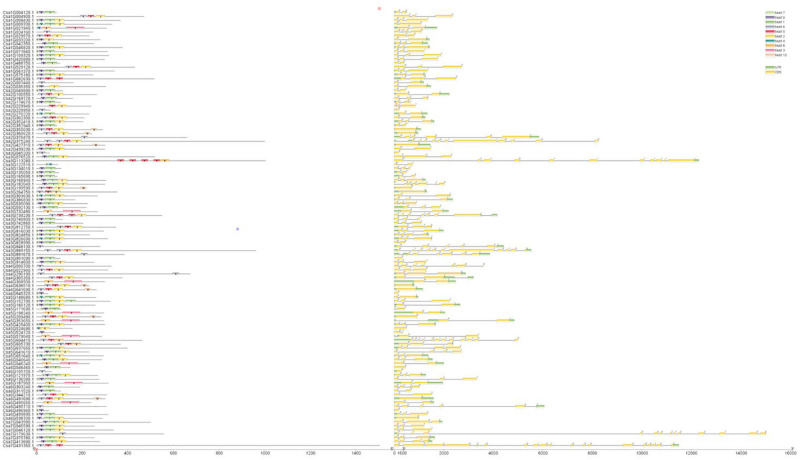
Conserved protein motifs and gene structure of *CsMYB* genes in cucumber. The left column shows conserved motifs in all the proteins encoded by *CsMYB* gene. There are 10 different color boxes with numbers 1–10 indicted 10 subclasses defined by conserved motifs. The right column shows exons, introns, and UTRs (untranslated regions) structures with yellow boxes, single lines and green boxes, respectively. Introns phases 0, 1 and 2 are indicated by numbers 0, 1 and 2, respectively. The length of each *CsMYB* gene can be estimated using the scale at the bottom.

**FIGURE 4:**
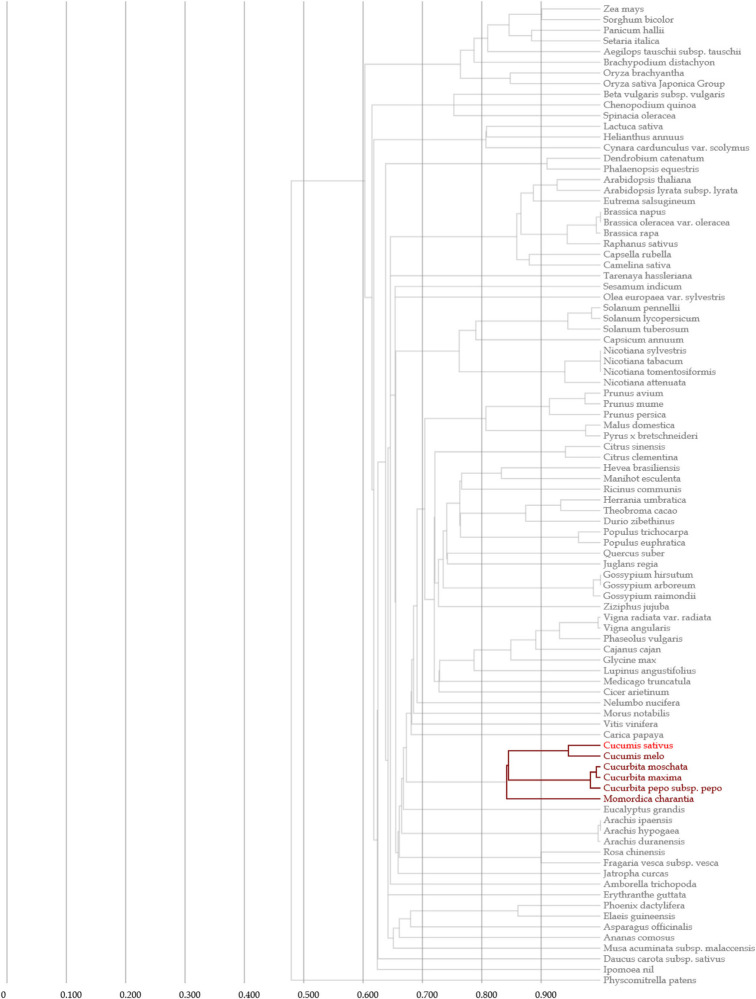
Phylogenetic tree for cucumber and other species based on MYB gene sequences from Gcorn database.

Clustal V was applied to generate multiple sequence alignment of the conserved structural domains of all cucumber *CsMYB* genes (Figure 1). Results were used to construct a phylogenetic tree (Figure 2), illustrating distribution and extent of conserved structural elements (Figure 3, Supplementary Table S1, and Supplementary Figure S2). As shown in Figure 3, the number of exons in *CsMYB* gene family members varied from 1 to 19 (18 with one exon, 34 with two exons, 41 with three exons, 6 with four exons, 1 with five exons, 3 with six exons, 3 with seven exons, 3 with eleven exons, 1 with twelve exons, 1 with thirteen exons and 1 with nineteen exons). Intron-exon structure of *CsMYB* genes was also analyzed after exton identification (Supplementary Figure S4). The results showed that exon 1 (133 bp) and exon 2 (130 bp) appeared to be more consistent in length, while exon 3 was more variable (31–850 bp). Phylogenetic analysis of the predicted CsMYB protein sequences revealed differences between cucumber and *Arabidopsis*, with eight sub-groups in common. Two cucumber subgroups were missing from *Arabidopsis*; ten sub-groups from Arabidopsis were absent in the cucumber genome, indicating *CsMYB* genes may evolve or be lost in a given taxon, following divergence. Some cucumber CsMYB proteins were clustered into *Arabidopsis* functional clades, which provided an excellent reference to explore the putative functions of the cucumber *CsMYB* genes. The comparative evolutionary analysis between *Arabidopsis* and cucumber suggests both species are derived from a common ancestor, no longer extant, and represent independent paths defined by duplication, divergence and loss of gene family members (Figure 4).

The obvious redundancy of *CsMYB* gene at the level of amino acid sequence is partly due to the similar molecular functions of *CsMYB* gene, but different biological phenotypes can still be displayed by these loci when mutated, due to different temporal or spatial expression characteristics. Studies have shown that homologous members of this gene family are not redundant in terms of plant development (Jin and Martin, 1999).

### Distribution and Collinearity Analysis of *CsMYB* Genes in Cucumber

**FIGURE 5:**
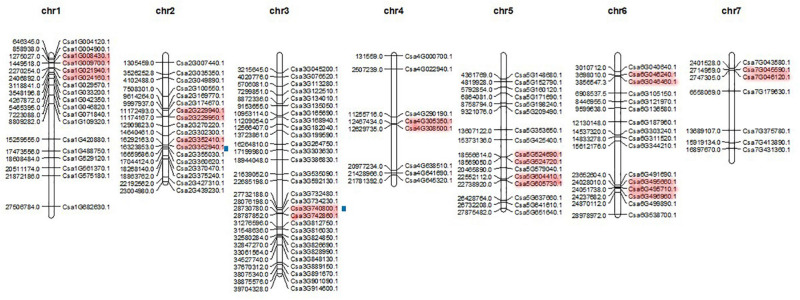
The chromosomal distribution of *CsMYB* genes positions. Chromosomal positions of all the *CsMYB* genes are indicated by the full name of the genes (all the genes assigned in Table 1). The red color regions in chromosomes indicate tandem duplication; Blue squares along chromosome indicate segmental duplication.

Members of the CsMYB gene family were mapped to all seven chromosomes with Mapchart (Figure 5). Results showed that chromosome 3 had the largest number of gene family members, 29 genes, while chromosome 7 had the fewest number of genes, containing only seven. The other five chromosomes 1, 2, 4, 5 and 6 each contained 18, 18, 8, 16, and 16 gene family members, respectively. The chromosome distribution of *CsMYB* gene family members is non-regular and does not correlate with overall chromosome length.

To further analyze divergence of *CsMYB* gene family members in cucumber, the tandem repeat events and fragment repeat events of the gene family were analyzed by using KAKS_Calculator2.0. In this analysis, if two or more genes are present within 200 kb, the elements are considered a tandem repeat event. In total, 23 *CsMYB* genes were involved in 11 tandem repeats as follows: (*Csa1G008430.1* & *Csa1G009700.1*, *Csa1G021940.1* & *Csa1G024160.1*, *Csa2G229940.1* & *Csa2G229950.1*, *Csa2G352410.1* & *Csa2G352940.1*, *Csa3G740800.1* & *Csa3G742860.1*, *Csa4G305350.1* & *Csa4G308500.1*, *Csa5G524690.1* & *Csa5G524720.1*, *Csa5G604410.1* & *Csa5G605730.1*, *Csa6G046240.1* & *Csa6G046460.1*, *Csa6G495660.1* & *Csa6G495710.1* & *Csa6G496960.1*, *Csa7G045590.1* & *Csa7G046120.1*). Gene family fragment duplication events were determined by BLASTP, which showed that only one pair of genes (*Csa3G740800.1*, *Csa2G352940.1*) had experienced fragment duplication. Based on the results above, it is inferred that tandem repeat events play an important role in the amplification of gene families rather than duplication of gene fragments.

**FIGURE 6:**
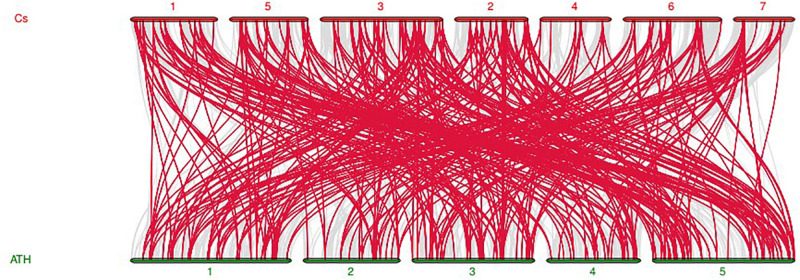
Synteny analysis of *MYB* genes between cucumber and *Arabidopsis thaliana.* The red lines indicate *MYB* collinear genes, and the gray indicated the collinear genes of both species. Cs represents cucumber, while ATH represents *Arabidopsis thaliana*.

In order to uncover the linkage relationships of *CsMYB* genes throughout the cucumber genome, collinearity analysis was carried out between cucumber and *Arabidopsis thaliana* MYB genes (Figure 6) showing that high homology of the MYB gene family existed between the two species. Twenty-five pairs of genes showed collinearity and conserved linkage relationships (Supplementary Figure S3).

### Analysis of *cis*-Elements of *CsMYB* Genes in Cucumber

**FIGURE 7:**
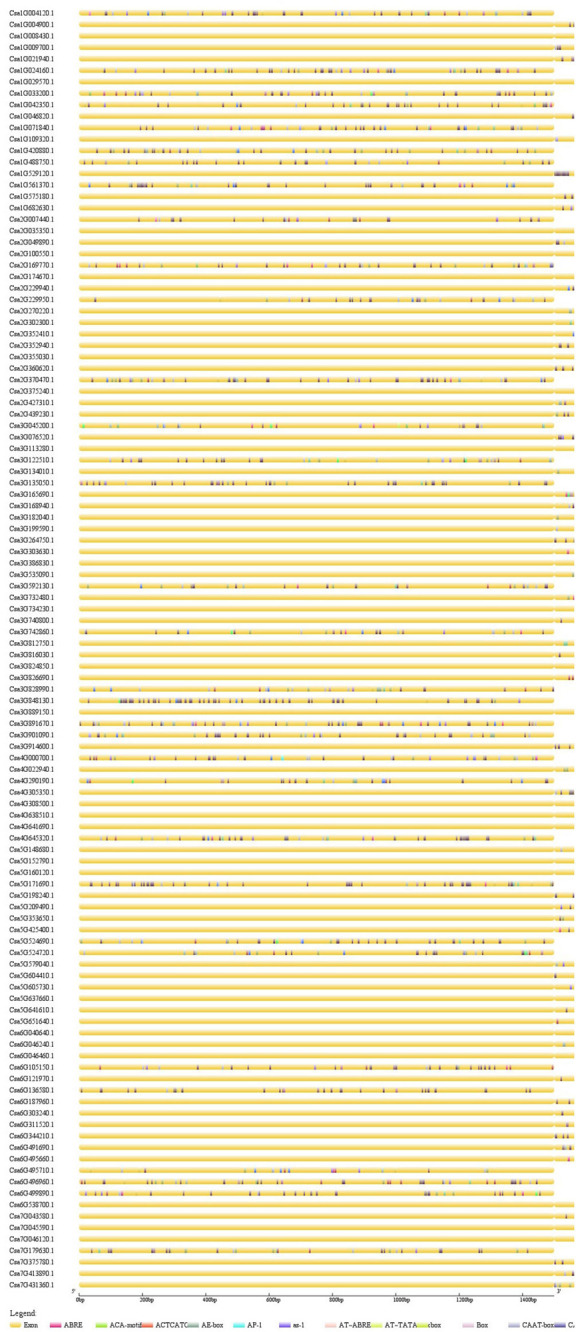
Analysis of *cis*-elements in the upstream regions of *CsMYB* genes. Predicted *cis*-elements in CsMYB promoters. Promoter sequences (–1500 bp) of 112 *CsMYB* genes were analysis by PlantCARE for further identification of regulatory elements. The upstream length to the translation start site can be inferred according to the scale at the bottom.

In order to study associated *cis*-elements of *CsMYB* promoters, the sequences (1.5 kb) upstream of *CsMYB* coding sequence were selected from cucumber genome, and then were submitted to PlantCARE for further identification of regulatory elements. Using the online site Genetic Structure Display Server (GSDS), visualization all regulatory elements was conducted involving TF binding sites in defense and stress responses or defense responses (Figure 7). Three genes, *Csa6G538700, Csa1G021940*, and *Csa5G641610* which had shown higher expression level in responses of cucumber to *M. incognita*, showed specific differences in the upstream *cis*-elements associated with the homolog in question. All three genes show associated upstream sequence that contains the *cis*-element G-box (TACGTG) and *cis*-element ABRE (ACGTG). The former sequence is associated with light-regulated gene expression; the latter is involved in regulation of gene expression by abscisic acid.

### Expression Analysis of MYB Gene Detected From Transcriptome

**FIGURE 8:**
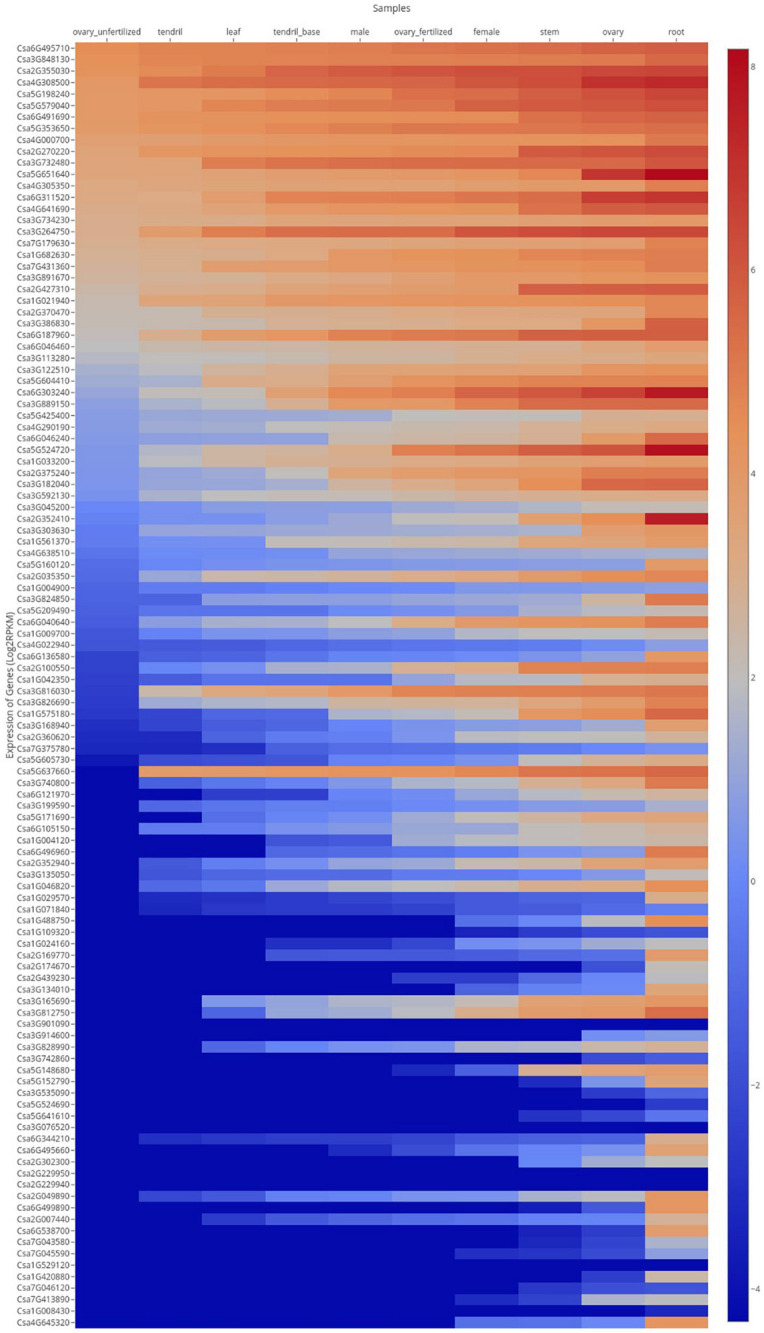
Expression profiles of *CsMYB* genes in different tissues or organs. FPKM values of *CsMYB* genes were transformed by log2 and a heatmap was constructed using the cucumber genome database HEATMAP to draw the heatmap online. Red color indicates a high level. Blue color indicates a low level of gene expression.

In order to analyze tissue-specific expression of the *CsMYB* gene family in cucumber, expression levels of gene family members were assayed in 10 tissues and organs obtained by PRJNA80169 (Figure 8). Results showed that most of the genes were expressed in all the different tissues sampled, with similar level of expression between tissues. Some genes, such as *Csa6G495710*, *Csa3G848130*, *Csa2G355030*, *Csa4G308500*, *Csa5G198240*, *Csa5G579040*, *Csa6G491690*, and *Csa5G353650*, were highly expressed in all 10 tissues and organs. Expression of *Csa3G076520*, *Csa2G229940*, *Csa2G229950*, *Csa1G529120*, and *Csa1G008430* were low in almost all tissues. *Csa6G303240*, *Csa5G524720*, *Csa5G160120*, *Csa6G136580*, *Csa3G168940*, *Csa6G496960*, *Csa2G007440*, *Csa3G824850*, *Csa1G029570*, *Csa1G488750*, *Csa2G169770*, *Csa5G152790*, *Csa5G605730*, *Csa3G740800*, *Csa6G344210*, *Csa6G495660*, *Csa2G049890*, *Csa6G499890*, *Csa6G121970*, *Csa3G134010*, *Csa1G420880, Csa6G538700*, *Csa4G645320*, and *Csa2G3522410* (21.4%) were only highly expressed in the root, showing lower levels of expression in other tissues and organs.

**FIGURE 9:**
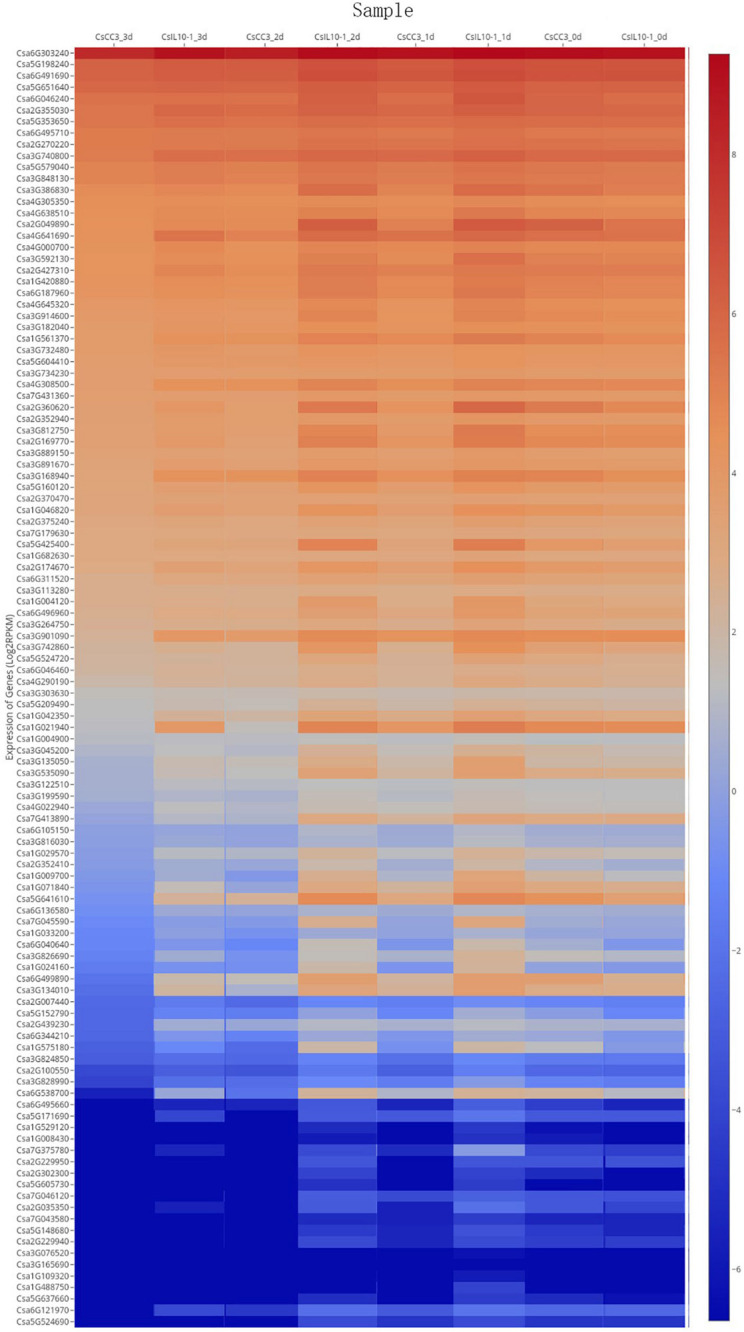
Expression profile of *CsMYB* genes through time after infection with *M. incognita* in resistant and susceptible cucumber lines, IL10-1 and CC3. Expression profile of *CsMYB* genes in response to the inoculation of *M. incognita* on published data RNA-seq SRP125669 (Wang et al., 2018). The color scale represents relative gene expression levels.

The post-RKN-inoculation transcriptome data extracted from resistant IL10-1 and susceptible CC3 lines were acquired using SRP125669 to ascertain whether members of the *CsMYB* gene family were subject to altered regulation during susceptible or resistant response to RKN (Figure 9). Six genes were randomly picked to validate the RNA-seq data. Additionally, to gain more insight into the role of *CsMYB* genes in the response to RKN in cucumber, relative real-time qPCR was performed to evaluate the transcript abundance of these *CsMYB* genes previously identified as differentially regulated during the resistant response to *M. incognita* (Supplementary Figure S5), which showed the similar expression trends with their RNA-seq data, respectively.

The homolog *Csa6G303240* was highly expressed in both resistant and susceptible cucumber genotypes, but at different time points, suggesting that expression of this gene was not specifically correlated with resistance to RKN in cucumber. Further comparative analysis of the transcriptomes from RKN-resistant and susceptible genotypes revealed that the MYB gene family member, *Csa5G641610*, shown to affect expression of the core gene in resistance to RKN, *LTP*, gradually decreased in CC3, while in IL10-1, transcript levels initially increased, then decreased. The expression level of *Csa5G641610* was analyzed, similar to the expression patterns of *Csa1G021940*, and *Csa6G538700*. Thus, it is speculated that *Csa6G538700*, *Csa1G021940*, and *Csa5G641610* may regulate gene *LTP* to some extent, further affecting resistance to RKN in cucumber.

## Discussion

### Organization and Characterization of the Cucumber *CsMYB* Gene Family

The MYB family is the largest TF gene family in plants and has been shown to play an important role in secondary metabolite formation, sexual differentiation, cell differentiation, plant morphogenesis, and biotic and abiotic stress (Jung et al., 2003; Hsu et al., 2005; Guo et al., 2013; Li et al., 2018). The MYB TFs are ubiquitous to all plant species and have been surveyed in several species with sequenced genomes, such as *Arabidopsis* (Riechmann et al., 2000), *grape* (Matus et al., 2008), *poplar* (Wilkins et al., 2009), *soybean* (Du et al., 2012), *maize* (Dias et al., 2003). Additionally, 55 members of the cucumber *R2R3MYB* gene family were identified and characterized from annotated genes in the 26682 cucumber genome (Li et al., 2012). In the current study, 112 *CsMYB* genes were identified in the last and previous versions of the genome assembly of the cucumber “Chinese Long” (v3). Compared to the draft genome sequence of *Cucumis sativus* var. *sativus* L. used by Li et al. (2012), the reference genome sequence has been improved, and therefore, our 112 *CsMYB* genes family, included those 55 *R2R3MYB* genes in which 36 genes show one to one correspondence (Supplementary Table S2).

The 112 *CsMYB* genes family was consist of 39 1RMYB, 69 R2R3-MYB, 3 R1R2R3-MYB and 1 4RMYB according to the number and type of MYB domains. All the details of the gene structure, conserved motifs, and *cis*-element were analyzed and illustrated. Structural analysis found that the lengths of exons 1 and 2 are very conserved, similar to previous results reported for cucumber *R2R3MYB* work (Li et al., 2012). However, in our study, exon 1 and 2 were not as highly conserved, perhaps because we investigated all the MYB genes instead of just the subclass, *R2R3MYB*, and the other subclasses of MYB genes increased the variation in structural conservation. All 112 *CsMYB* genes have been located on seven chromosomes with uneven distribution. The largest number of homologs are found chromosome 3 and the smallest number on chromosome 7 (Figure 5). The locations are generally consistent with the more limited results in a previous study (Li et al., 2012). It is interesting to note that after the correspondence was identified between the published MYB gene (*R2R3-MYB*_v1) (Li et al., 2012) and *CsMYB*_v2 (Supplementary Table S2), two inversions were found in chromosome 5 and 7, (*Csa 5G148680.1*, *Csa5G152790.1* and *Csa5G160120.1* on chr.5, *Csa7G046120.1* and *Csa7G045590.1* on chr.7), compared with the chromosomal locations of Li et al. (2012). These chromosomal inversions might due to the differences between the improved cucumber genome and the old version released in 2009, which been reported (Lou et al., 2013).

Although whole genome duplication events occur commonly in many angiosperms, especially the recent gene duplications which are crucial for the rapid expansion and evolution of gene families (Cannon et al., 2004; Taylor and Raes, 2004), whole-genome duplication events and tandem duplications are rare in cucumber genome (Huang et al., 2009). In this study of the *CsMYB* gene family, tandem repeat events were detected more frequently than duplication of gene fragments in further analysis of the divergence of *CsMYB* gene family members, thus duplication may be the main driving force in the amplification of gene families.

### Expression Analysis of *CsMYB* Genes Expressed in Response to *M. incognita*

Studies show that selection of favorable interactions between stress tolerant genes and their TFs can be an effective method to improve the comprehensive stress tolerance (Sun et al., 2010). The *MYBS1* gene of *Arabidopsis thaliana* is involved in salt stress of plants in response to various plant hormones (such as auxin, gibberellin, jasmonic acid, salicylic acid). In cucumber, the TF, *Csa1G021940*, is the homolog of *MYBS1*, and also responds to various phytohormones, suggesting that, similar to its homolog in Arabidopsis, this gene could condition a response to biological stress in cucumber, such as RKN infection (Gutierrez et al., 2009; Molinari et al., 2014; Zhao et al., 2018). In Arabidopsis, the *MYB67*TF (cucumber homolog, *Csa6G538700)* is involved in cell differentiation (Gheysen and Mitchum, 2011), thus in cucumber, perhaps the homolog may also affect the differentiation of giant cells which would hinders the development of *M*. *incognita*, thereby causing resistance to *M*. *incognita* in IL10-1. Finally, in Arabidopsis, *AT3G30210.1* (cucumber homolog, *Csa5G641610*) is a TF that responds to abscisic acid (ABA) which in cucumber, could inhibit cell elongation, affecting giant cell elongation, thereby enhancing the resistance to RKN. The results from our previous study of physiological observations of abnormal development of giant cells (GCs) between IL10-1 (resistant line) and CC3 (susceptible line) indicated that a series of reactions may occurred after the infection of RKN in the resistant line. Also, multi-omics study showed high levels of expression of the *LTP* gene which has been identified as the “hub gene” involved in lipid transport in GCs membranes and receives signals stimulated by nematodes (Wang et al., 2019).

In this study, the expression data for all 112 *CsMYB* genes in the two lines, IL10-1 and CC3, contrasting for RKN resistance were obtained directly from NCBI by SRP125669 (Wang et al., 2018) at 0, 1, 2 and 3 days after inoculation (dpi) with *M*. *incognita*. Further comparative analysis of the transcriptomes from IL10-1 (RKN-resistant) and CC3 (RKN-susceptible) revealed that three genes (*Csa5G641610*, *Csa1G021940*, and *Csa6G538700*) in *CsMYB* gene family, showed the same expression pattern where they initially increased, then decreased in IL10-1, but only decreased through time in the susceptible line, CC3. It further affected the expression of *LTP*, which assumed to be the core gene in response to RKN (Wang et al., 2018). In our previous comparative study on transcriptional events, combining with the physiological responses that occurs in resistant line IL10-1 and susceptible line CC3 during *M*. *incognita* infection, potential effector-targeted host genes were identified, while their regulation network was illuminated in the IL10-1 against *M*. *incognita*. Through the construction of a co-expression network, 2 out of 8 genes with the highest edges, considered the core genes of the network and assumed to have consequential roles in the resistant response against *M*. *incognita* perhaps as a result of abnormal development of GCs, were related to plant lipid transfer proteins (LTPs). One of the two most significantly induced *LTP* genes, which had the most edges, was considered as the hub gene in the resistance to RKN (Wang et al., 2018). More and more studies have indicated that LTPs are associated with plant resistance and defense (Thoma et al., 1994; Jung et al., 2003), for instance, LTPs can inhibit α-amylase, thereby contributing to resistance to biotic stress in wheat (Oda et al., 1997). However, reports on the relationships between LTPs and plant nematodes are still limited. As a result, further studies are necessary to elucidate the exact role of LTPs in plant resistance to nematodes. Studies in Arabidopsis show that the Myb-type DNA-binding transcriptional factor, MYB96, positively regulated the downstream gene *LTP3* to mediate freezing and drought stress (Guo et al., 2013). Whether there is a correlation between MYB TFs and LTP genes during plant-pathogen interaction process remains unknown.

## Conclusion

In summary, this paper carried out the basic bioinformatics analysis including the characterization, the phylogenetic analysis and evolutionary analysis of the 112 *CsMYB* genes newly identified in cucumber. Additionally, comparative analysis of the transcriptomes discovered three *CsMYB* genes (*Csa5G641610*, *Csa1G021940*, and *Csa6G538700*) might positively regulate predicted hub gene *LTP* (*Csa6G410090*) in resistance to RKN in cucumber. The exact relationship between these three MYB TFs and the LTP gene during the plant-pathogen interactions, as well as the mechanisms how *CsMYB* genes regulate LTP to work will require further study.

## Data Availability Statement

Publicly available datasets were analyzed in this study. This data can be found at NCBI under the accession PRJNA80169, https://www.ncbi.nlm.nih.gov/search/all/?term=PRJNA80169.

## Author Contributions

JC and MJ designed and managed the project. QL, JL, and XW handled the samples and performed the experiments. QL and CC performed the data analyses and wrote the manuscript. All authors reviewed and approved this submission.

## Conflict of Interest

The authors declare that the research was conducted in the absence of any commercial or financial relationships that could be construed as a potential conflict of interest.

## Footnotes

1 http://cucurbitgenomics.org/

2 http://pfam.xfam.org/

3 http://skylign.org/

4 http://web.expasy.org/protparam/

5 http://cello.life.nctu.edu.tw/

6 www.plant.osakafu-u.ac.jp/∼kagiana/gcorn/p

7 http://bioinformatics.psb.ugent.be/webtools/plantcare/html/

8 http://gsds.cbi.pku.edu.cn/

9 https://www.ncbi.nlm.nih.gov/sra/

10 http://cucurbitgenomics.org/

## Supplementary Material

Supplementary Figure 1 Special Hidden Markov Model (HMM) of *CsMYB* gene in cucumber. The special HMM of *CsMYB* gene in cucumber was built according to the MYB HMM (PF00249) from PFam with the parameter (*E* ≤ 1.2 × 10^–8^). Then the logo of special model was visualizated online by SKylign.

Supplementary Figure 2 Sequence logo of conserved Motif 1 to Motif 10.

Supplementary Figure 3 Circos diagram of *CsMYB* genes in cucumber. Twenty-five pairs of *CsMYB* genes showed collinearity and linkage relationships in cucumber.

Supplementary Figure 4 Exon length distribution analysis of the cucumber MYB genes. **(A)** Exon length values were analyzed using Boxplot. Each box represents the exon size range in which 50% of the values for a particular exon are grouped. The mean value is shown as a dotted line and the median as a continuous line. The number of exons in *CsMYB* gene family members varied from 1 to 19. **(B)** First, second and third exon lengths distribution of *CsMYB* genes using 3D Scatter Plot.

Supplementary Figure 5 Relative RT-qPCR assay of six CsMYB family genes randomly picked from RNA-seq data in CC3 and IL10-1 at 0, 1, 2, 3 days post inoculation with *M. incognita*. **(A–F)** represented *Cs1RMYB-15*, *Cs2RMYB-7*, *Cs2RMYB-4*, *Cs2RMYB-65*, *Cs1RMYB-18*, and *Cs2RMYB-63*, respectively. The default expression value for each gene was Actin gene. The default expression value for each gene was from 0 dpi plants. Error bars indicate the standard deviation from the mean (three replicates).

Supplementary Table 1 The sequence and characterize of motif.

Supplementary Table 2 Correspondence between the published MYB gene (*R2R3-MYB*_v1) in cucumber (Li et al., 2012) and identified genes (*CsMYB*_v2) in cucumber.

Supplementary Table 3 Primers Sequencs of the three random picked genes.
