# Supplementary material for: Identification and Expression Analysis of the CsMYB Gene Family in Root Knot Nematode-Resistant and Susceptible Cucumbers
Source: Front Genet. 2020 Dec 3;11:550677. doi: 10.3389/fgene.2020.550677 (PMC7744742; doi:10.3389/fgene.2020.550677)
Supplement: Supplementary Figure 1 — Special Hidden Markov Model (HMM) of CsMYB gene in cucumber. The special HMM of CsMYB gene in cucumber was built according to the MYB HMM (PF00249) from PFam with the parameter (E ≤ 1.2 × 10^–8^). Then the logo of special model was visualizated online by SKylign. [file Data_Sheet_1.docx]

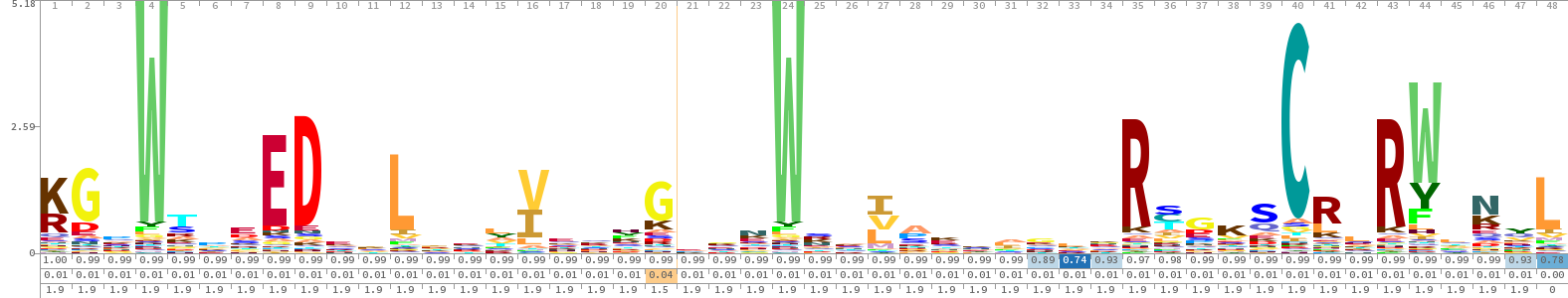


**Figure S1 Special Hidden Markov Model (HMM) of** ***CsMYB* gene in cucumber.** The special HMM of *CsMYB* gene in cucumber was built according to the MYB HMM (PF00249) from PFam with the parameter (E < = 1.2 × 10-8). Then the logo of special model was visualizated online by SKylign.


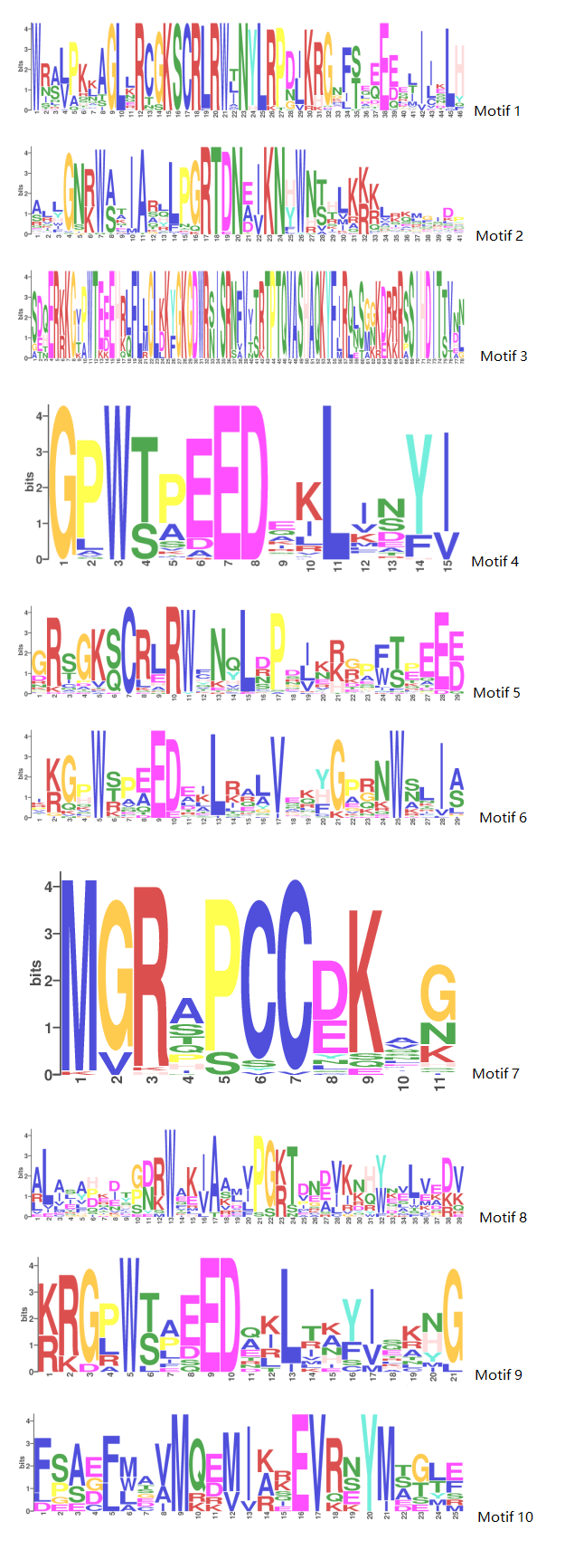


**Figure S2 Sequence logo of conserved Motif 1 to Motif 10**


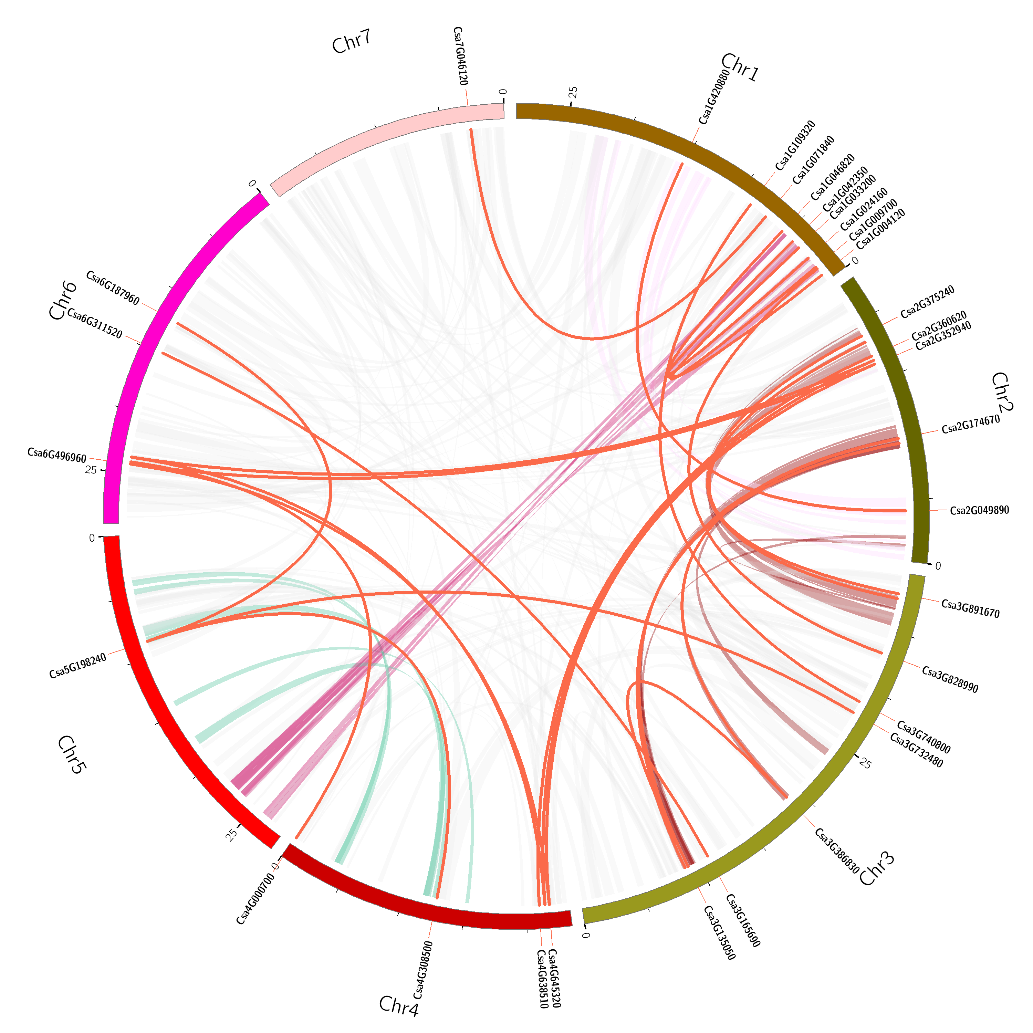


**Figure S3 Circos diagram of *CsMYB* genes in cucumber.** Twenty-five pairs of *CsMYB* genes showed collinearity and linkage relationships in cucumber


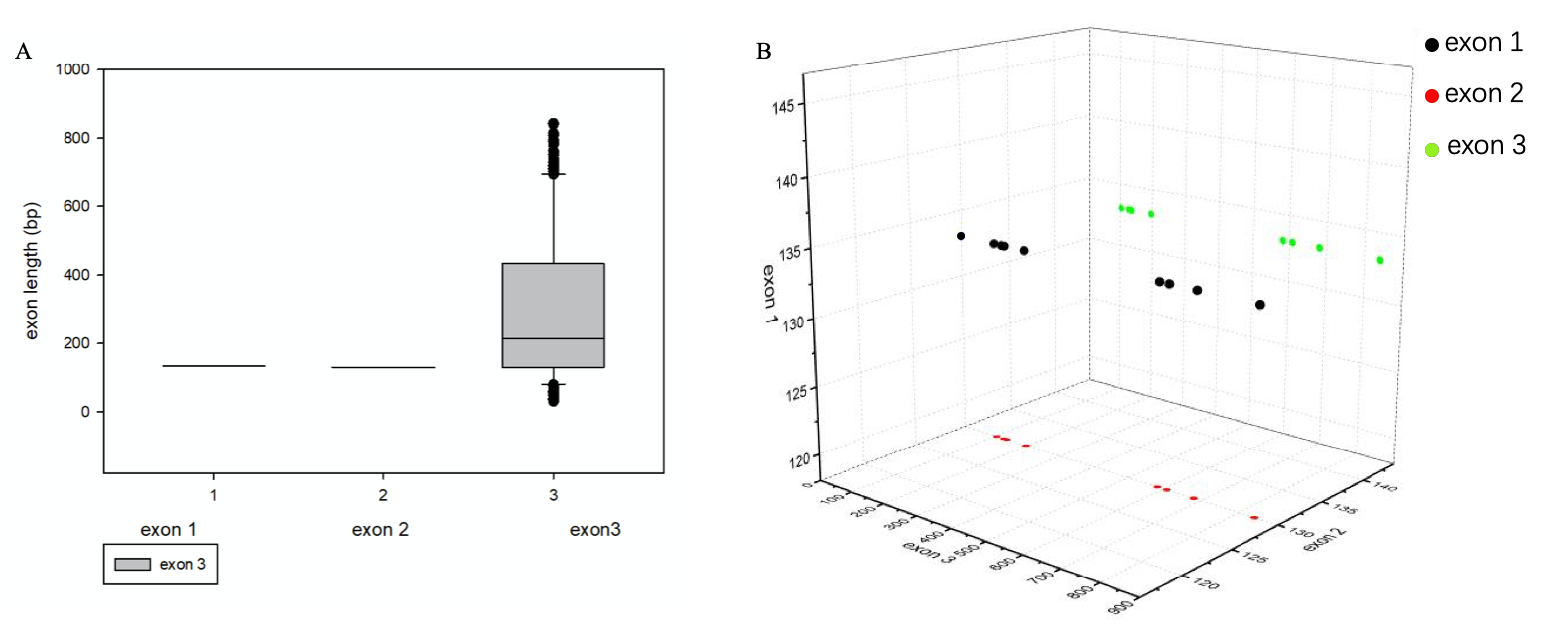


**Figure S4 Exon length distribution analysis of the cucumber MYB genes.** (A) Exon length values were analysed using Boxplot. Each box represents the exon size range in which 50% of the values for a particular exon are grouped. The mean value is shown as a dotted line and the median as a continuous line. The number of exons in *CsMYB* gene family members varied from 1 to 19. (B) First, second and third exon lengths distribution of *CsMYB* genes using 3D Scatter Plot.

**
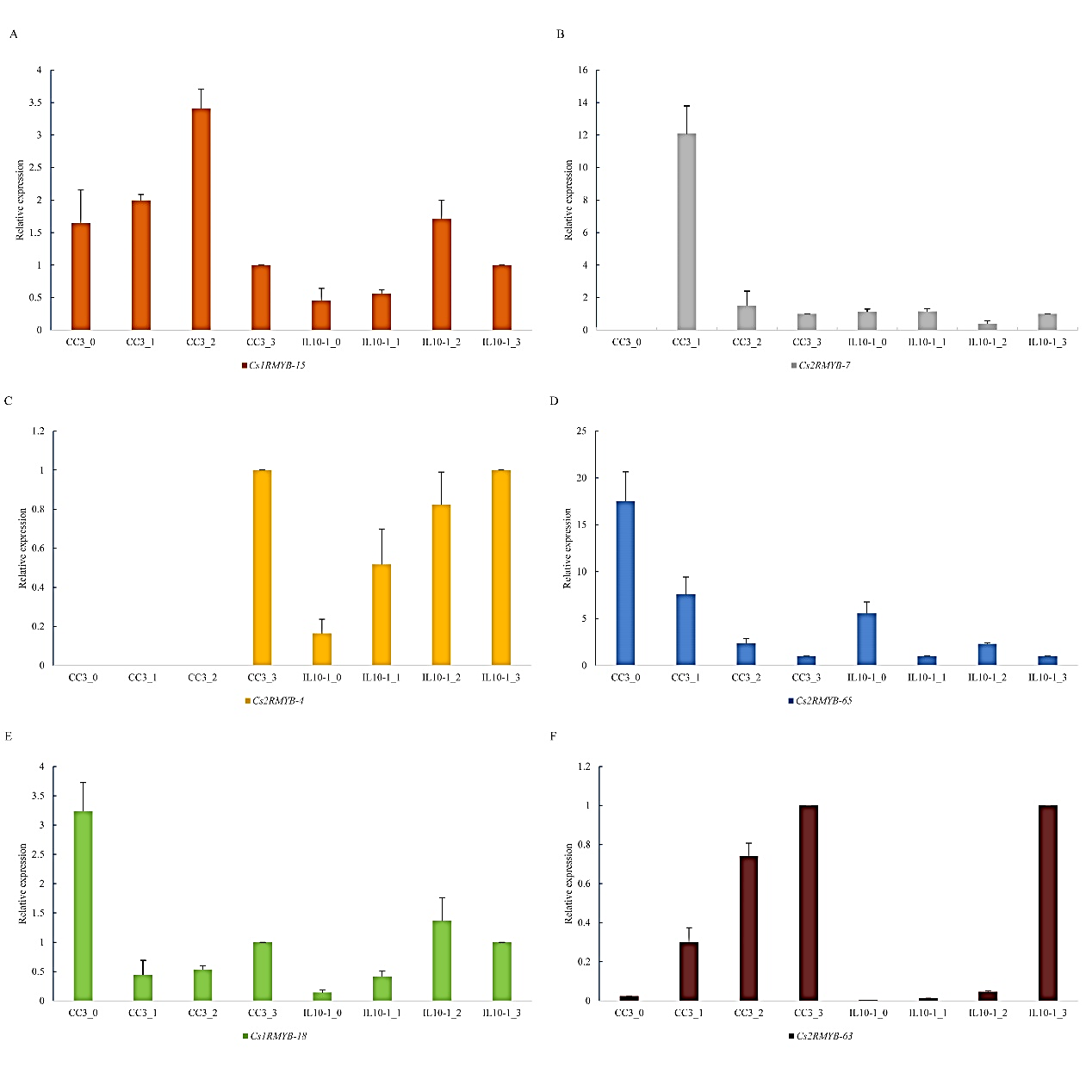
Figure S5 Relative RT-qPCR assay of six CsMYB family genes randomly picked from RNA-seq data in CC3 and IL10-1 at 0, 1, 2, 3 days post inoculation with *M. incognita*. (**A), (B), (C), (D), (E), and (F) represented *Cs1RMYB-15*, *Cs2RMYB-7*, *Cs2RMYB-4*, *Cs2RMYB-65*, *Cs1RMYB-18*, and *Cs2RMYB-63*, respectively. The default expression value for each gene was Actin gene. The default expression value for each gene was from 0 dpi plants. Error bars indicate the standard deviation from the mean (three replicates).

Table S1 The sequence and characterise of motif

| Motif | sequence | width | sites | E-value |
| --- | --- | --- | --- | --- |
| 1 | W[RN][AS][LV]PK[KL]AGL[LK]RCGKSCRLRW[TLI]NYLRP[DN][IL] | 46 | 50 | 2.0e-1645 |
|  | KRGNF[ST]E[EQ]E[ED]E[LIT]I[IL]XLH |  |  |  |
| 2 | [AS]LLGN[RK]W[AS]TIAR[LQ]LPGRTDN[EAD][IV]KN[HY]WN | 41 | 50 | 3.0e-1094 |
|  | [TS][HT]LK[KR][KR]L[RK][KQ]MGIDP |  |  |  |
| 3 | S[DE][QH]ER[KR]KG[IVT][PA]WTE[ED]EH[RK][LQ]FL[LM]GL | 78 | 9 | 6.2e-438 |
|  | [KD]K[YF]GKGDWR[SN]ISRNFV[IVT][TS]RTPTQVASHAQK |  |  |  |
|  | YF[IL]R[QL][LN]S[GM][GNK]K[DE][RK][RK]RSSIHDIT[TS]V[ND][LN] |  |  |  |
| 4 | GPW[TS][PA]EEDE[KI]LI[NS]Y[IV] | 15 | 50 | 1.9e-419 |
| 5 | GRSGK[SQ]CRLRW[CF]N[QY]L[DNR]PD[ILV][KN][RKH][GR] | 29 | 40 | 7.2e-556 |
|  | P[FW][TS][EP]EE[DE] |  |  |  |
| 6 | I[KR]G[PH]W[STR]P[EA]EDE[IK]L[RK]A[LA]VE[KQ][YHF]GP | 29 | 34 | 6.2e-387 |
|  | [RG]NW[SN]LI[AS] |  |  |  |
| 7 | MGRAPCC[DE]KX[GN] | 11 | 44 | 1.7E-255 |
| 8 | AL[AI]XA[HDP]X[DI]T[GP][DN][RK]WAK[IV]AA[LM] | 39 | 17 | 2.3E-167 |
|  | [VI]PG[KR]TDN[DEA][VI]K[NK][HQ][YW]X[EV]LVE[DK][VK] |  |  |  |
| 9 | [KR]RG[PL]W[TS][AP]EED[AQ][KI]LT[AK][YF][IV]XK[HN]G | 21 | 12 | 9.2E-60 |
| 10 | F[SP][AS][EDG][FL][MLW]A[VA]MQ[ED][MV]I[AKR][KR]E | 25 | 8 | 1.3E-50 |
|  | VR[NES]YM[AST][GT][LT][EF] |  |  |  |

Table S2 Correspondence between the published MYB gene (*R2R3-MYB*_v1) in cucumber (Li et al., 2012) and identified genes (*CsMYB*_v2) in cucumber

| *R2R3-MYB*_v1 | *CsMYB*_v2 | *R2R3-MYB*_v1 | *CsMYB*_v2 |
| --- | --- | --- | --- |
| Csa012797 | Csa5G148680.1 | Csa010143 | Csa6G491690.1 |
| Csa001869 | Csa7G413890.1 | Csa011496 | Csa2G355030.1 |
| Csa002643 | Csa3G816030.1 | Csa012498 | Csa5G171690.1 |
| Csa002717 | Csa3G824850.1 | Csa012824 | Csa5G152790.1 |
| Csa003349 | Csa7G046120.1 | Csa015272 | Csa3G303630.1 |
| Csa003581 | Csa2G360620.1 | Csa016676 | Csa4G022940.1 |
| Csa003827 | Csa1G046820.1 | Csa017164 | Csa1G109320.1 |
| Csa004708 | Csa3G740800.1 | Csa017450 | Csa3G826690.1 |
| Csa005181 | Csa1G008430.1 | Csa017539 | Csa5G160120.1 |
| Csa005219 | Csa1G004120.1 | Csa017970 | Csa4G305350.1 |
| Csa007739 | Csa6G121970.1 | Csa019830 | Csa3G076520.1 |
| Csa008131 | Csa5G425400.1 | Csa022057 | Csa3G168940.1 |
| Csa008771 | Csa3G199590.1 | Csa013585 | Csa7G045590.1 |
| Csa009345 | Csa4G645320.1 | Csa008474 | Csa6G105150.1 |
| Csa009412 | Csa4G638510.1 | Csa018305 | Csa5G209490.1 |
| Csa009484 | Csa4G641690.1 | Csa000784 | Csa6G538700.1 |
| Csa009573 | Csa5G651640.1 | Csa019344 | Csa7G375780.1 |
| Csa009688 | Csa5G637660.1 |  |  |

Table S3 Primers Sequencs of the three random picked genes

| Primer Name | Primer Sequence (5’-3’) |
| --- | --- |
| ACTIN-F | ATTGTTCTCAGTGGTGGTTCTAC |
| ACTIN-R | CCTTTGAGATCCACATCTGCT |
| *Cs1RMYB-15-F* | ATACTTCGACTGGGATTTCG |
| *Cs1RMYB-15-R* | GTTCGGAACATTCAAGTCAC |
| *Cs1RMYB-18-F* | GACTGCTGAAGAAGACAGAC |
| *Cs1RMYB-18-R* | TCTTTTGCCTTTTTCTGTTGC |
| *Cs2RMYB-4-F* | TCGATAAGTTTGGGAAAGGG |
| *Cs2RMYB-4-R* | CCACCGCCATTATTAACTGA |
| *Cs2RMYB-7-F* | CAGGTTGAGATGGCTTAACT |
| *Cs2RMYB-7-R* | TCTGGATGTGGTTATTTGGG |
| *Cs2RMYB-63-F* | AATCATTGACGTTCACCGTA |
| *Cs2RMYB-63-R* | TCAAGACCCTGAGCAATAAG |
| *Cs2RMYB-65-F* | TAATGCCAGGAAGAACAGAC |
| *Cs2RMYB-65-R* | TTGACCTCTCAATTTCCCTG |
